# Distal gastric tube resection with preservation of the right gastroepiploic artery for gastric tube cancer: a case report

**DOI:** 10.1186/s40792-021-01340-2

**Published:** 2021-12-20

**Authors:** Kohei Tajima, Hideo Shimada, Takayuki Nishi, Yutaro Kamei, Kazuo Koyanagi, Hiroyasu Makuuchi

**Affiliations:** 1 Department of Gastroenterological Surgery, Tokai University School of Medicine, 143 Shimokasuya, Isehara, Kanagawa 259-1193 Japan; 2 Department of Surgery, Tokai University Oiso Hospital, 21-1 Gakkyou, Nakagun, Oiso, Kanagawa 259-0198 Japan

**Keywords:** Gastric tube cancer, Distal gastric tube resection, Preservation of the right gastroepiploic artery, Less-invasive treatment, Long-term follow-up after esophagectomy

## Abstract

**Background:**

The incidence of gastric tube cancer is increasing because of improved survival rates in patients with esophageal cancer treated by esophagectomy. Total resection of the gastric tube is expected to be highly curative, but it is associated with a higher risk of severe postoperative complications. Herein we report a case of early gastric tube cancer that was successfully treated by distal gastric tube resection with preservation of the right gastroepiploic artery (RGEA).

**Case presentation:**

An 82-year-old man was diagnosed as having gastric tube cancer, B-12-O, Type 0-IIc, T1b, N0, M0, cStage IA (Japanese Classification of Gastric Carcinoma). Upper gastrointestinal endoscopy showed a Type 0-IIc lesion measuring 30 mm in length in the lower part of the gastric tube, and histopathological examination of biopsy specimens revealed the features of poorly differentiated adenocarcinoma. The primary lesion could not be identified by computed tomography, and there was no obvious lymph node metastasis or distant metastasis. Considering that total resection of the gastric tube would have been highly invasive and that the gastric tube cancer was at a relatively early stage, we performed distal gastric tube resection with preservation of the RGEA. The postoperative course was uneventful, and the patient was discharged on postoperative day 12. There has been no recurrence during the 17 months of follow-up.

**Conclusion:**

We successfully treated a patient with gastric tube cancer by distal gastric tube resection with preservation of the RGEA. This treatment strategy may be acceptable for patients with early gastric tube cancer without lymph node metastasis, considering the balance between the surgical invasiveness and curability of the tumor.

## Background

The incidence of gastric tube cancer is increasing because of improved survival rates in patients with esophageal cancer who are treated by esophagectomy [1, 2]. The principle of treatment for gastric tube cancer is resection of the stomach with lymph node dissection. However, total resection of the gastric tube is highly invasive, and is associated with a high risk of severe postoperative complications [3, 4]. Herein, we report a case of early gastric tube cancer that was successfully treated by distal gastric tube resection with preservation of the right gastroepiploic artery (RGEA).

## Case presentation

**Fig. 1:**
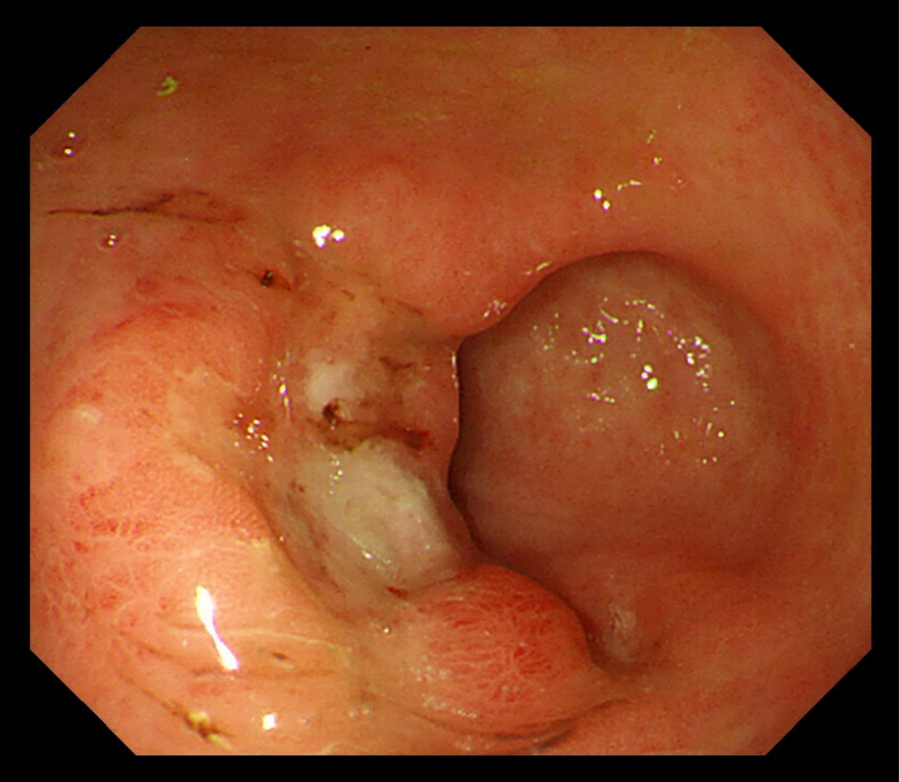
Preoperative upper gastrointestinal endoscopy findings. A Type 0-IIc lesion of 30 mm in length was found in the lower part of the gastric tube, and pathological examination of biopsy specimen revealed poorly differentiated adenocarcinoma

**Fig. 2:**
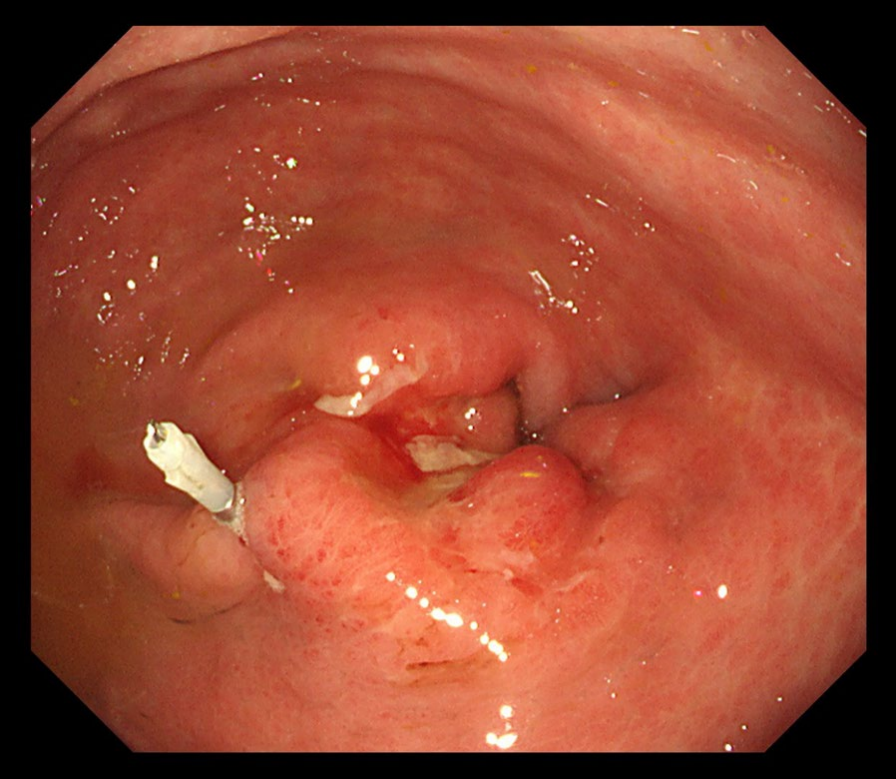
Endoscopic clipping. Marking clipping on the oral side of the tumor was performed to confirm the location and extent of the lesion

**Fig. 3:**
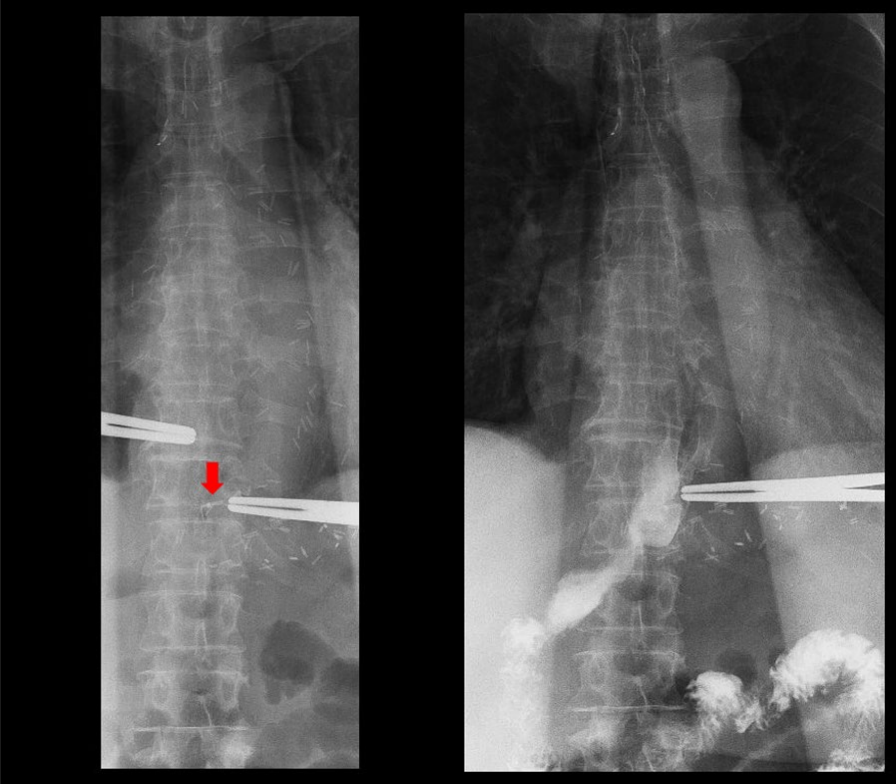
Upper gastrointestinal series findings. We reconfirmed the location of the clip and recognized that performing the distal gastric tube resection may be possible

**Fig. 4:**
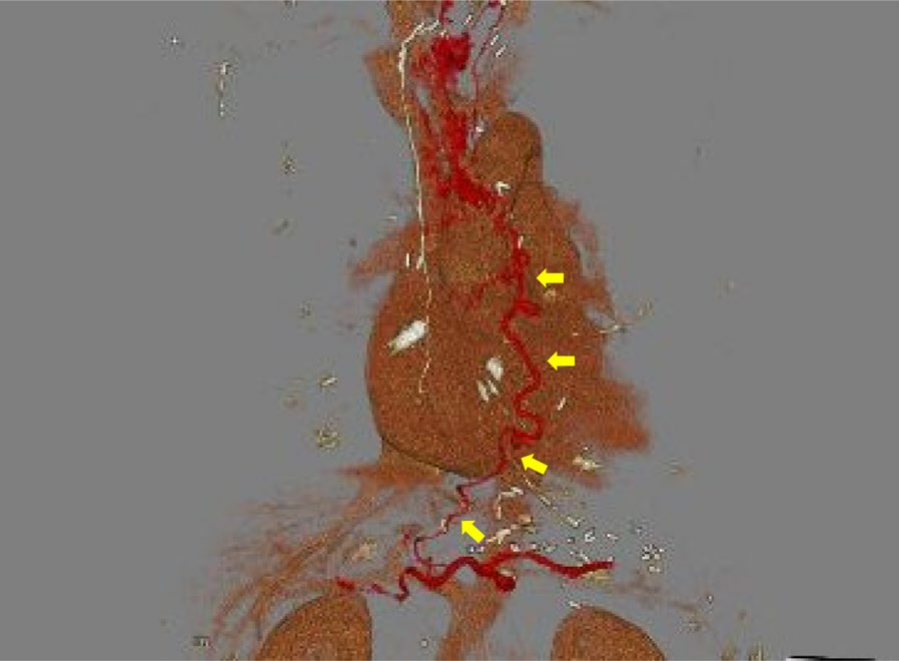
3D-CT angiography findings. The dominant region of the RGEA was distributed to the upper part of the gastric tube

**Fig. 5:**
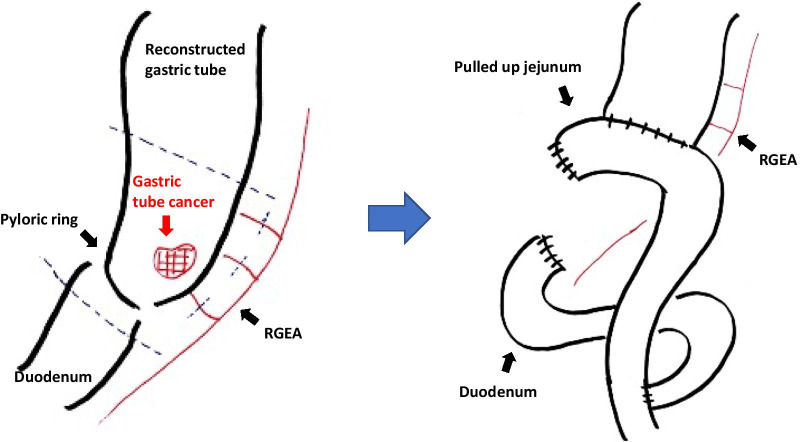
Schema of distal gastric tube resection with preservation of the right gastroepiploic artery and Roux-en-Y reconstruction. The dissection line of reconstructed gastric tube, duodenum and the branches of the RGEA (dotted line). (*RGEA* right gastroepiploic artery)

**Fig. 6:**
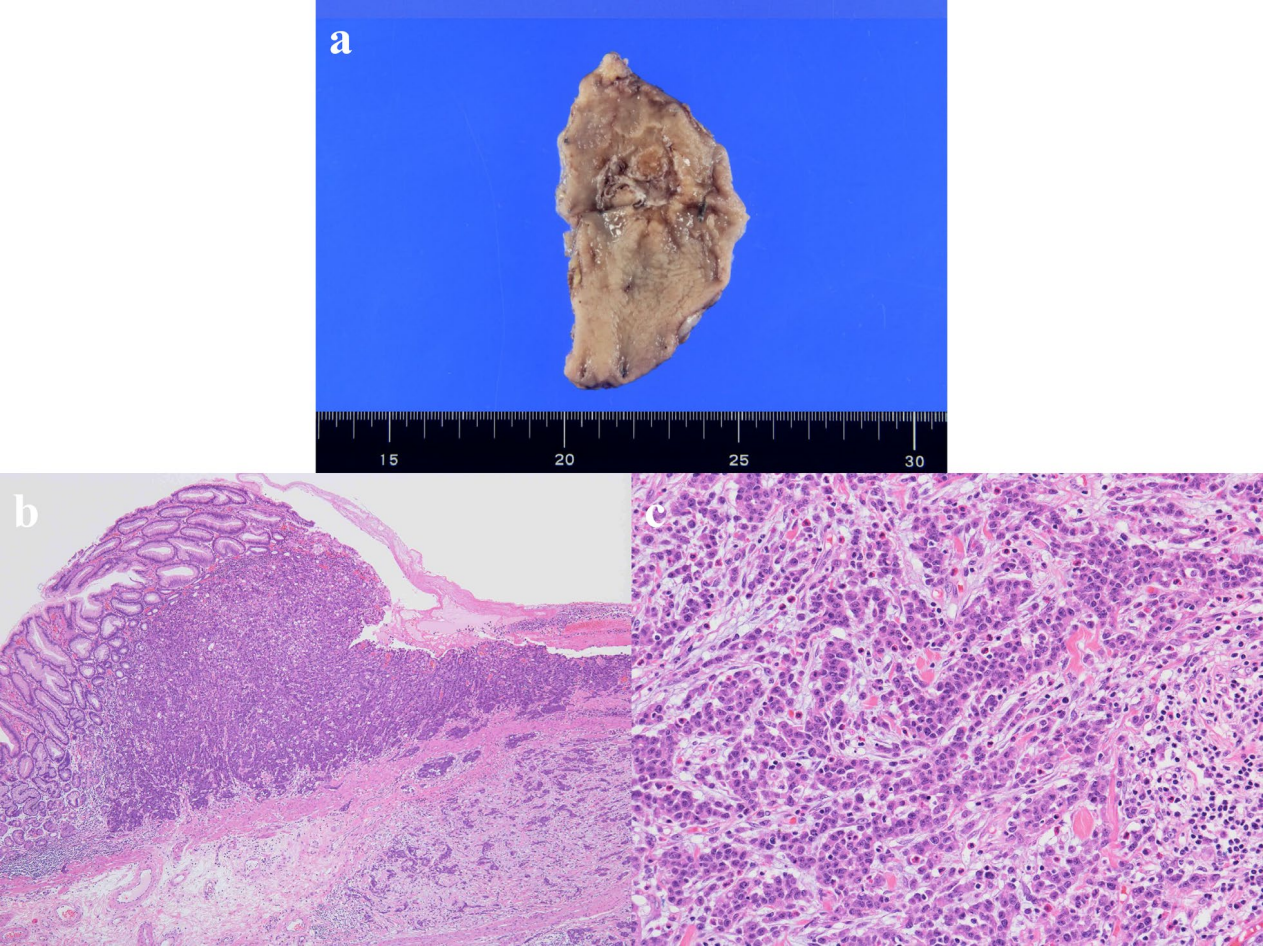
Pathological findings. Pathological examination of the resected material revealed poorly differentiated adenocarcinoma with submucosal invasion. **a** Macroscopic examination showed gastric tube cancer of 27 × 25 mm. **b** Hematoxylin and eosin staining results, ×4. **c** Hematoxylin and eosin staining results, ×20

An 82-year-old man with no symptoms visited our hospital for routine postoperative follow-up examinations after esophagectomy for esophageal cancer. At the age of 70, he had undergone thoracic esophagectomy trans right thoracic approach with gastric tube reconstruction via the antethoracic route. The follow-up upper gastrointestinal endoscopy showed a Type 0-IIc lesion measuring 30 mm in length in the lower part of the gastric tube (Fig. 1), and histopathological examination of biopsy specimens revealed features consistent with poorly differentiated adenocarcinoma. The primary lesions could not be detected by computed tomography (CT), and there was no obvious lymph node metastasis or distant metastasis. We made the diagnosis of gastric tube cancer, B-12-O, Type 0-IIc, T1b, N0, M0, cStage IA (Japanese Classification of Gastric Carcinoma). Ideally, total resection of the gastric tube would have been indicated for the lesion, as it was a poorly differentiated adenocarcinoma with submucosal invasion. However, because of the advanced age of the patient (82 years), high surgical invasiveness of total resection of the gastric tube, and relatively early stage of the diagnosed cancer, we considered distal gastric tube resection with preservation of the RGEA as a potentially suitable surgical strategy for the patient. To examine the feasibility of performing such a surgical procedure, we repeated the upper gastrointestinal endoscopy and clipped the oral side of the tumor to confirm the location and extent of the lesion (Fig. 2). Thereafter, an upper gastrointestinal series was performed to reconfirm the location of the clip, and we recognized that distal gastric tube resection could be surgically performed (Fig. 3). 3D-CT angiography showed that the dominant region of supply of the RGEA was distributed towards the upper part of the gastric tube (Fig. 4). Therefore, we determined that preservation of the RGEA would be required to perform distal gastric tube resection. A midline incision was made along the previous surgical scar, the abdomen was opened, dissection was performed around the gastric tube, and the RGEA was exposed. The RGEA supplying blood to the gastric tube was identified and preserved. The extent of the tumor was determined by palpation of the endoscopic clip, and elevation of the small intestine up to the chest wall was also confirmed. The dissection line of the gastric tube was determined to allow complete resection of the tumor, and several vessels branching from the RGEA and entering the gastric wall in the resection area were ligated. We did not perform the lymph node dissection and the sentinel node navigation surgery because of no perigastric lymph nodes recognized during the surgery and the risk of conversion to total resection of the gastric tube due to RGEA injury. The stomach on the oral side and duodenum on the anal side of the tumor were divided using an automatic suturing device, and the distal gastric tube resection was completed. The duodenal stump was closed as a blind end. The small intestine was pulled up to the chest wall and an antecolic Roux-en-Y reconstruction was completed (Fig. 5). The operation time was 252 min, and the blood loss was 44 ml. The postoperative course was uneventful, and the patient was discharged on postoperative day 12. The histopathological diagnosis was gastric tube cancer, B-12-O, pType 0-IIa, por2 > tub1, pT1bN0M0, pStage 1A (Japanese Classification of Gastric Carcinoma) (Fig. 6) [5]. No adjuvant chemotherapy was administered. The patient has remained free of recurrence until now, 17 months since the surgery.

## Discussion

In recent years, the survival rates of patients with esophageal cancer have improved with advances in the diagnostic methods, surgical outcomes, and adoption of multidisciplinary treatment approaches. With these advances the incidence of gastric tube cancer after esophagectomy has increased to 2.9–3.5% [2, 6, 7].

The principles of treatment of gastric tube cancer are often extrapolated from those of treatment of gastric cancer, namely, resection of the stomach with lymph node dissection. Total resection of the gastric tube is expected to be highly curative, but is associated with a higher risk of severe postoperative complications due to the extremely invasive nature of the surgical procedure. Therefore, endoscopic submucosal dissection (ESD), endoscopic mucosal resection (EMR), and distal gastric tube resection with preservation of the RGEA have been adopted as less invasive treatments. Several recent reports have demonstrated the usefulness of less invasive treatments for early gastric tube cancer. We previously reported a case of EMR for early gastric tube cancer [8]. Yura et al. reported distal gastric tube resection with preservation of the main trunk of the RGEA and right gastric artery for a case of early gastric tube cancer that had invaded the duodenum [9]. They also summarized that only five previous reports described the procedure. Because in the majority of cases of gastric tube cancer, the lesion is located in the lower part of the gastric tube (68%) [6], there might be a relatively large number of candidates for distal gastric tube resection with preservation of the RGEA. Gastric tube was reconstructed via antethoracic route in our case; however, this procedure is considered to be possible regardless of the previous reconstruction route, if distal gastric tube is present in the abdominal cavity [9].

Although there have been few previous reports, distal gastric tube resection with preservation of the RGEA is considered as an acceptable procedure, considering the balance between the surgical invasiveness and curability of the tumor. Mucosal tumors in which ESD is not indicated, and submucosal tumors without lymph node metastasis may be good indications. Even in early stage cancers, it is extremely important to ensure that there is no obvious lymphadenopathy on CT. However, greater caution is needed in selecting candidates who have advanced cancer, because in such cases, the procedure may be less likely to be curative than total resection of the gastric tube. Therefore, preoperative tumor staging is extremely important before such surgical procedures are selected, and these surgical procedures are not recommended in cases with advanced cancer because of the higher incidence of regional lymph node metastasis.

In our case, preoperative judgement was needed to determine the feasibility of distal gastric tube resection with preservation of the RGEA. First, preoperative recognition of the exact localization of the tumor was essential. We performed upper gastrointestinal endoscopy and placed a clip on the oral side of the tumor to confirm the location and extent of the lesion. Then, an upper gastrointestinal series was performed to reconfirm the location of the clip. Based on the findings of the above examinations, we determined that distal gastric tube resection with antecolic Roux-en-Y reconstruction might be a rational surgical treatment procedure. Second, preoperative evaluation of the dominant region of supply of the RGEA was mandatory. Such as intraoperative ICG fluorescence imaging may be useful for evaluation of remnant gastric tube blood flow. In this case, we performed 3D-CT angiography, which revealed that the dominant region of supply of the RGEA was distributed to the upper part of the gastric tube. Based on these findings, we decided to perform distal gastric tube resection with preservation of the RGEA, to maintain the blood perfusion of upper part of the gastric tube. Saito et al. reported the blood supply for 5 cm of proximal region of the gastric tube was adequate by ICG fluorescence [10]. Therefore, it is difficult to perform the distal gastric tube resection without preservation of RGEA. On the other hand, although we successfully completed the resection and reconstruction without injury the RGEA, ICG angiography may be useful as intraoperative recognition of blood vessel status.

Inevitably, early-stage gastric tube cancer has a better prognosis than advanced gastric tube cancer. In some case reports, gastric tube cancer was diagnosed in the long-term after esophagectomy, as in our case [10, 11]. Overlap between esophageal cancer and gastric cancer is known to be common. Periodic follow-up endoscopy after esophagectomy is essential, and early detection and treatment of gastric tube cancer by long term surveillance can improve the prognosis [12].

The incidence of gastric tube cancer in patients of advanced age could be expected to increase because of the improved survival rates in patients with esophageal cancer treated by esophagectomy. However, only a limited number of elderly patients might be able to tolerate total gastric tube resection. On the other hand, partial resection of the gastric tube may be a viable alternative procedure that can be tolerated even by elderly patients. Therefore, we believe that distal gastric tube resection with preservation of the RGEA will become more important as a less-invasive surgery in the future.

## Conclusion

We successfully treated a patient with gastric tube cancer by distal gastric tube resection with preservation of the RGEA. This treatment strategy is acceptable for early gastric tube cancers without regional lymph node metastasis and should be considered in older patients, as it offers a balance between surgical invasiveness and curability of the tumor.

## Data Availability

All data generated during this study are included in this published article.

## Abbreviations

CT: Computed tomography
RGEA: Right gastroepiploic artery
ESD: Endoscopic submucosal dissection
EMR: Endoscopic mucosal resection
